# Research progress of ultrasound in accurate evaluation of cartilage injury in osteoarthritis

**DOI:** 10.3389/fendo.2024.1420049

**Published:** 2024-08-15

**Authors:** Huili Zhang, Eryu Ning, Lingfeng Lu, Jing Zhou, Zhiqiang Shao, Xing Yang, Yuefeng Hao

**Affiliations:** ^1^ Orthopedics and Sports Medicine Center, The Affiliated Suzhou Hospital of Nanjing Medical University, Suzhou, China; ^2^ Gusu School, Nanjing Medical University, Suzhou, China

**Keywords:** cartilage, osteoarthritis, ultrasonography, knee joint, elasticity imaging techniques

## Abstract

Osteoarthritis (OA) is a prevalent cause of joint algesia, loss of function, and disability in adults, with cartilage injury being its core pathological manifestation. Since cartilage damage is non-renewable, the treatment outcome in the middle and late stages of OA is unsatisfactory, which can be minimized by changing lifestyle and other treatment modalities if diagnosed and managed in the early stages, indicating the importance of early diagnosis and monitoring of cartilage injury. Ultrasound technology has been used for timely diagnosis and even cartilage injury treatment, which is convenient and safe for the patient owing to no radiation exposure. Studies have demonstrated the effectiveness of ultrasound and its various quantitative ultrasound parameters, like ultrasound roughness index (URI), reflection coefficient (R), apparent integrated backscatter (AIB), thickness, and ultrasound elastography, in the early and accurate assessment of OA cartilage pathological changes, including surface and internal tissue, hardness, and thickness. Although many challenges are faced in the clinical application of this technology in diagnosis, ultrasound and ultrasound-assisted techniques offer a lot of promise for detecting early cartilage damage in OA. In this review, we have discussed the evaluation of ultrasonic cartilage quantitative parameters for early pathological cartilage changes.

## 1 Introduction

**Figure 1:**
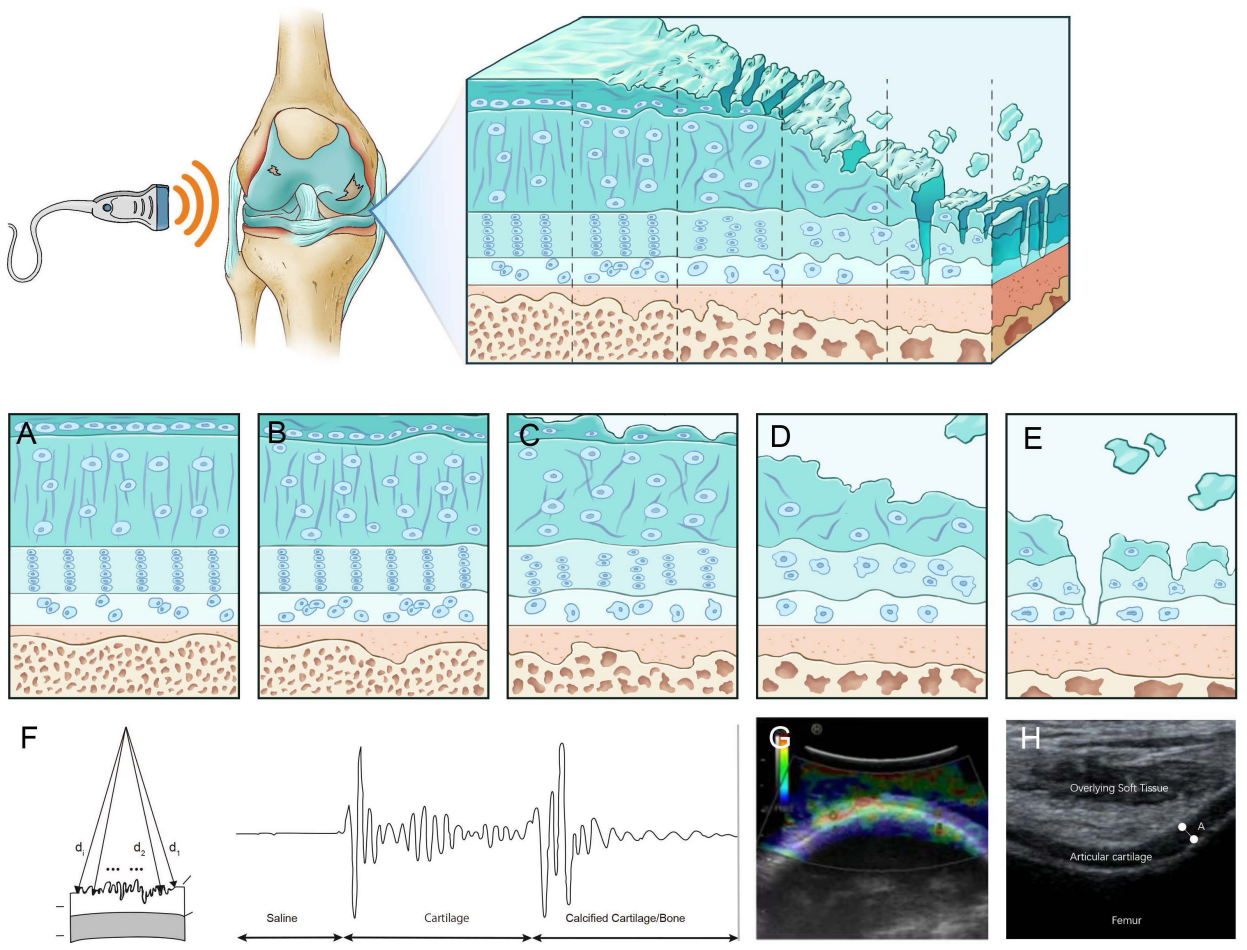
Ultrasound assessment of OA cartilage injury diagram **(A)** In the early stages of cartilage damage, URI, R, and IRC can identify the surface roughness of the cartilage. **(B)** R, AIB can reflect the abnormal collagen network organization and composition in the early stage of cartilage injury. **(C)** Rbone, IRC, and AIB are related to the surface area of trabecular bone, which can reflect the degeneration of subchondral bone. **(D)** Elastic ultrasound can distinguish between normal cartilage and pathological cartilage. **(E)** Ultrasound can detect cartilage thickness and defect degree. **(F)** Calculate a schematic diagram of quantitative ultrasound parameters. d_i_,d_1_, and d_2_ are the lengths from the transducer to the surface. **(G)** Cartilage schematic diagram under ultrasound elastography. Reprinted from Clinical Anatomy, Vol. 32, Sonoelastography of the knee joint, Akkaya M, Cay N, Gursoy S, Simsek ME, Tahta M, Dogan M, et al., Pages 99-104, doi: 10.1002/ca.23300 (5), this diagram has been authorized. **(H)** Ultrasound measurement of cartilage thickness schematic diagram. A represents the thickness of cartilage.

Osteoarthritis (OA) is a common and prevalent skeletal degenerative condition, where cartilage injury and degree of damage are regarded as the prime pathological changes incurred (1). Changes in the shape and structure of the cartilage surface and the components of cartilage in joints are essential symptoms and diagnostic bases for evaluating cartilage injury. Additionally, the severity of cartilage injury is an essential reference for various scoring systems, including the International Cartilage Repair Society (ICRS). Applying cartilage repair treatments like self-chondrocyte implantation and self-osteochondral transplantation, as well as OA management medicines, necessitates a more accurate and objective evaluation of articular cartilage and subchondral bone integrity (2, 3). Therefore, accurate and sensitive evaluation of cartilage injury, real-time monitoring, changes in cartilage status assessment, and timely adoption of corresponding treatment measures are vital factors in OA diagnosis and treatment, which are pivotal for preventing late-stage complications (4) (**Figure 1**).

**Figure 2:**
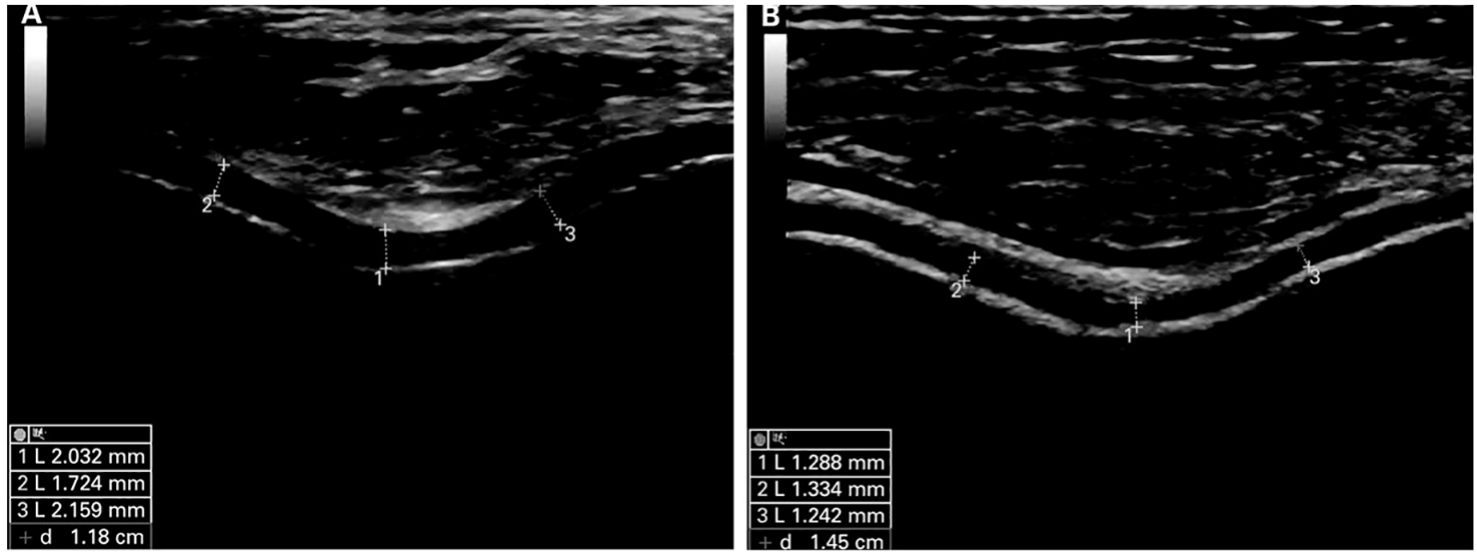
Ultrasound images of normal and damaged cartilage tissue in mode B. **(A)** is classified as normal cartilage. Articular cartilage shows hyperechoic, sharply defined interfaces. **(B)** is classified as pathological cartilage. Articular cartilage appears thinner and shows less defined interfaces. Reprinted from Annals of the Rheumatic Diseases, Vol. 68, Ultrasound Validity in the Measurement of Knee Cartilage Thickness, Naredo E, Acebes C, Moller I, Canillas F, de Agustin JJ, de Miguel E, et al., Pages 1322-1327, doi: 10.1136/ard.2008.090738 (10), this diagram has been authorized.

Ultrasound (US) is a safe, non-radiation, low-cost, and widely used technique for diagnosing musculoskeletal diseases and can give information about synovitis, joint effusion, periarticular soft tissues, and bony cortical abnormalities in peripheral OA joints (4, 6–8). Due to the high content of water and the absence of inner acoustic interfaces, the cartilage presents as hypoechoic or anechoic bands. Divided by two sharp hyperechoic interfaces of the cartilage-bone interface and synovial space-cartilage interface (9), the main characteristics of healthy patient joint cartilage are low echo or anechoic and clear cartilage-bone interface and synovial fluid-cartilage interface. OA patients exhibit unevenly scattered echo bands on the surface or middle part of the tissue due to surface and internal degeneration such as decreased water content and fibrous degeneration. Mechanical damage results in joint cartilage damage and loss (**Figure 2**). Lately, studies have demonstrated that US-assisted technology can quantitatively detect cartilage changes and can disclose early cartilage pathologies or evaluate cartilage damage, which can measure cartilage thickness (6, 11, 12). In early osteoarthritis, the loss of proteoglycans and the destruction of surface collagen lead to fibrosis and softening of the soft bone surface (13, 14). Quantitative ultrasound parameters can provide information on surface fibrosis of articular cartilage, reflecting the destruction of surface collagen and the loss of proteoglycans, which helps to distinguish between normal and degenerative articular cartilage in the early stages of osteoarthritis (15, 16). Ultrasound elastography, as a new US imaging method, can detect articular cartilage softening before structural changes in knee osteoarthritis(KOA) and distinguish pathological cartilage from normal cartilage in the early stage of osteoarthritis (17, 18). It can detect changes in the hardness of articular cartilage before structural changes in knee osteoarthritis, which helps to achieve the goal of early diagnosis of OA. Cartilage thickness is an important indicator for describing the development and progression of osteoarthritis. Detecting cartilage thickness and the degree of cartilage damage is crucial for evaluating the progression and treatment response of OA (13, 19). Quantitative ultrasound parameters, ultrasound elastography, and ultrasound detection of soft bone thickness play a crucial role in the early diagnosis and treatment of OA and are crucial for preventing late complications and slowing down disease progression. Systematic articles on US-based evaluation of cartilage injuries still need to be included. This review aims to provide information about the US application in assessing OA cartilage injury and puts forward some suggestions for progress in this field.

## 2 Application of ultrasound in cartilage injury assessment

### 2.1 Quantitative ultrasound parameters have the potential to become measurement tools for the quantitative analysis of articular cartilage

The quantitative ultrasound parameters for evaluating cartilage injury assessment include the ultrasound roughness index (URI), reflection coefficient (R), cartilage-bone interface reflection coefficient (Rbone), apparent integrated backscatter (AIB), and integrated ultrasonic reflection coefficient (IRC). The early pathological manifestations of OA cartilage injury include surface fibrillation (13) and tissue swelling (14), due to the reduction in proteoglycan on the surface of cartilage in joints and the destruction of the surface collagen network. A study reported that the above-stated quantitative ultrasound parameters of cartilage could sensitively detect mechanical degeneration, roughness changes of the cartilage surface and spontaneous fibrous fibrillation, enzymatic destruction of the surface collagen network, and degeneration of the subchondral bone, with an ability to distinguish between normal and degenerative articular cartilage in the initial stages of OA (**Table 1**).

**Table 1:** Measurement methods for partial quantitative ultrasound parameters reported in the literature.

Parameter	Equation
Ultrasound roughness index (13)	$URI=\sqrt{\frac{1}{m}\sum_{i=1}^{m}{\left(di-\langle d\rangle \right)}^{2}}$
Ultrasound reflection coefficient (14)	$R=\frac{1}{m}\sum_{i=1}^{m}\frac{A_{si}}{A_{iref}}$
Cartilage-bone interface reflection coefficient (14)	$R_{bone}=\frac{1}{m}\sum_{i=1}^{m}\frac{A_{bi}}{A_{iref}}$
Apparent integrated backscatter (13)	$AIB=\frac{1}{\Delta f}\int_{\Delta f}10\log_{10}\langle \frac{{\left|A_{b}\left(f,z\right)\right|}^{2}}{{\left|A_{0}\left(f,z\right)\right|}^{2}}\rangle df$
Integrated ultrasonic reflection coefficient (13)	$IRC=\frac{1}{\Delta f}\int_{\Delta f}10\log_{10}\langle \frac{{\left|A_{1}\left(f,z\right)\right|}^{2}}{{\left|A_{0}\left(f,z\right)\right|}^{2}}\rangle df$

m is the sample length’s number of scan lines. di is the length from the transducer to the solution-cartilage boundary in the line i.〈d〉 is the average length from the transducer to the surface. The peak-to-peak amplitude of the ultrasonic RF signals that are reflected off the cartilage surface and the cartilage-bone contact, respectively, in line i is denoted by the letters Asi and Abi. Airef is the reference peak-to-peak amplitude measured from the solution-air interface at the same distance as Asi. Δf is the analyzed frequency range; Indices 0 and 1 refer to values obtained from the perfect reflector and sample, respectively. A(f, z) = amplitude spectrum of the pulse reflected at distance z from the transducer; Ab(f, z) = amplitude spectrum of the pulse backscattered at distance z from the transducer.

The URI can monitor the cartilage surface microstructure and describe the morphological changes, where R and AIB of the cartilage surface are sensitive to the change in collagen content and structure. Similarly, R is also used to describe the characteristics of cartilage tissue (20), and cartilage surface R depicts the acoustic parameter in cartilage enzyme degradation (21). Furthermore, the AIB is sensitive to alterations in the number and direction of the collagen network (22); a drop in R and IRC, as well as a rise in URI, can diagnose enhanced cartilage surface roughness. Similarly, a decreased R and IRC on the cartilage surface also depicts enzyme-induced surface collagen network degeneration. Besides cartilage health assessment, US has also been shown to be sensitive to subchondral bone degeneration (23). The R and IRC of the cartilage-bone interface were significantly correlated with the trabecular bone’s surface volume ratio and trabecular thickness. In the initial phases of OA, the bone around the joint is prone to change, including increased subchondral bone thickness, decreased subchondral trabecular bone mass, and the progression of calcified cartilage areas (24).

Moreover, quantitative ultrasound parameters are also helpful for accurately grading OA cartilage damage and viewing variations in the cartilage and internal tissues. Studies have demonstrated that increased URI is associated with an increased OA grade (14, 25, 26). With the progression of OA grading, the cartilage surface gets unequal and unpolished. Similarly, OA cartilage R decreases significantly compared to normal, whereas a decreased R significantly decreases with OA development. The increased surface roughness results in diffused reflection, reducing the echo amplitude (14). Additionally, with OA development, the cartilage softens, and the composition and framework of articular cartilage gradually change from the surface to the deep section. Since soft cartilage absorbs more transmission ultrasonic energy, the R-value decreases. As the OA stage increased, so did the R-value of the cartilage-bone interface, which was significantly higher than normal cartilage. Furthermore, the IRC was also strongly related to the early OARSI grade, where an increased IRC in OA was related to the R of the cartilage surface due to the destructive interference of incoherent waves scattered by surface fibrillation. An increased AIB might indicate abnormal organization and composition of the collagen network (16) since the AIB slope of early OARSI grading increased, whereas the AIB slope of degenerative cartilage samples was higher than that of healthy cartilage samples. The increased AIB slope in degenerated cartilage could be attributed to the collagen network rearrangement since the disorganized structure of diseased cartilage leads to greater backscatter than the deep vertical arrangement of fibers in normal cartilage (27).

The study reported that the cartilage surface R might be a more effective indicator than the URI and the cartilage-bone interface R to distinguish early OA grading (28). Many studies have also shown that the surface roughness index and R strongly correlate with the pathological evaluation of articular cartilage (14). In summary, quantitative ultrasound parameters can be used as a helpful assessment technique for quantitative articular cartilage assessment (14, 16, 29). They have also been applied for the quantitative diagnosis of cartilage lesions *in vivo* and *in vitro*, demonstrating the feasibility of *in vivo* US (**Table 2**).

**Table 2:** Study on quantitative ultrasound parameters in measuring cartilage injury.

Authors	Material	Transducer frequency (MHz)	Anatomical site	n	Acquisition of ultrasonic parameters	Usage
Saarakkala S, Toyras J, et al. (20)	Mechanical degradation and enzymatic degradation of the bovine knee joint	20	Patella osteochondral specimens	44	URI, R, and IRC	Quantitative ultrasound imaging can detect collagen damage and an increase in the surface roughness of articular cartilage.
Viren T, Saarakkala S, et al. (30)	Surgical repair or spontaneous healing of rabbit knee joint tissue	40	Repair-site osteochondral specimens	13	URI, R, AIB, and IRC	Ultrasound can evaluate the surface integrity and internal structure of repaired tissues.
Viren T, Saarakkala S, et al. (31)	Mechanically degraded bovine knee joint specimens	40	Knee joint cartilage	7	URI, R, AIB, and IRC	Ultrasound can evaluate the integrity of the cartilage surface.
Niu HJ, Wang Q, et al. (14)	Rabbit knee cartilage specimens after ACL surgery	55	MFC, LFC, MTP, and LTP	18	URI, R, and Rbone	Ultrasound can detect changes in URI and R after an ACL operation.
Liukkonen J, Hirvasniemi J, et al. (23)	Cadaver specimens without a history of joint disease	9	FAC	13	URI, R, R_bone_, AIB, and IRC	Ultrasound can evaluate the thickness and roughness of the cartilage surface.
Wang Q, Liu Z, et al. (32)	Rat knee joint cartilage	50	MFC, LFC, MTP, and LTP	14	URI, R	Ultrasound can detect the morphological and acoustic changes of knee joint cartilage.
Huang YP, Zhong J, et al. (33)	Total knee arthroplasty specimens of advanced knee osteoarthritis	25	Knee joint	10	URI, IRC	Ultrasound can measure the morphological changes at the junction of bone and cartilage.
Zhang J, Xiao L, et al. (34)	Porcine cartilage samples digested with trypsin and healthy control samples	15, 25	Porcine knee joint	36	IRC, AIB	Ultrasound can evaluate the integrity of the cartilage surface or the microstructure of the cartilage matrix.
Pastrama M, Spierings J, et al. (35)	Goats	31.25	Areas that articulate with the focal knee resurfacing implant and non-articulating areas	16	URI	Ultrasound can serve as a follow-up tool for evaluating cartilage quality.
Lye TH, Gachouch O, et al. (16)	Early human OA knee replacement specimens	40	MTP and LFC	26	AIB, IRC	Ultrasound can serve as a method for the early diagnosis and monitoring of osteoarthritis.

URI, ultrasound roughness index; R, ultrasound reflection coefficient; R_bone_, cartilage-bone interface reflection coefficient; AIB, apparent integrated backscatter; IRC, integrated ultrasonic reflection coefficient; ACL, anterior cruciate ligament; MFC, medial femoral condyle; LFC, lateral femoral condyle; MTP, medial tibial plateau; LTP, lateral tibial plateau; FAC, femoral articular cartilage.

### 2.2 Ultrasound elastography provides elastic information

#### 2.2.1 The main classification and application of ultrasound elastography

The World Federation of Ultrasound Medicine and Biology has defined it as strain and shear wave imaging according to the measurement of elastography, where the former depicts tissue deformation when the probe exerts pressure on the tissue along the propagation direction of the ultrasonic beam (including manual squeezing and acoustic radiation force pulse technique(ARFI)), while the latter is obtained by comparing the echo signals before and after compression (36). Strain imaging uses strain ratio to evaluate the deformation ability of tissue, where an increased strain ratio indicates softening, while acoustic elastography is based on shear wave technology (including transient elastography (TE) and ARFI), which excites the tissue to produce shear waves followed by measuring shear wave velocity. The hardness can be classified based on measuring the shear wave velocity, or Young’s modulus. The ARFI method does not depend on the compression applied to the surface and can be used to evaluate deeper-position organs (37). Ultrasound elastography uses color maps to evaluate the tissue’s deformability, where a change in color from blue to red indicates softening, overcoming the weakness of subjectivity of manual palpation, providing new elastic diagnostic information, expanding the scope of clinical application, could detect deep lesions and superficial masses, and has also been applied in cartilage injury (**Table 3**).

**Table 3:** The calculation method of tissue stiffness is evaluated by elastography technology (38).

Measured physical quantity	Method	Type of elastography	Indicators	Schematic diagram
Strain or Displacement	Strain imaging	Strain elastography	Strain ratio E/B size ratio	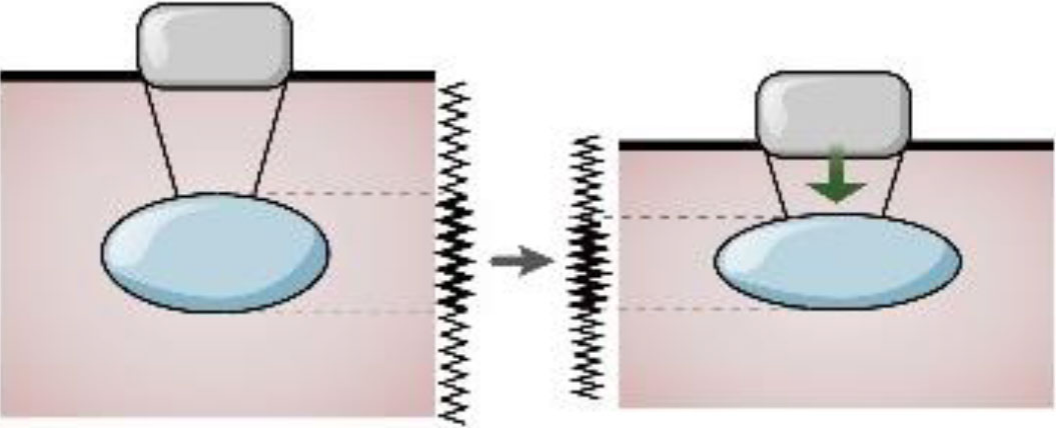
		Acoustic Radiation Force Impulse (ARFI) imaging	Displacement ratio E/B size ratio	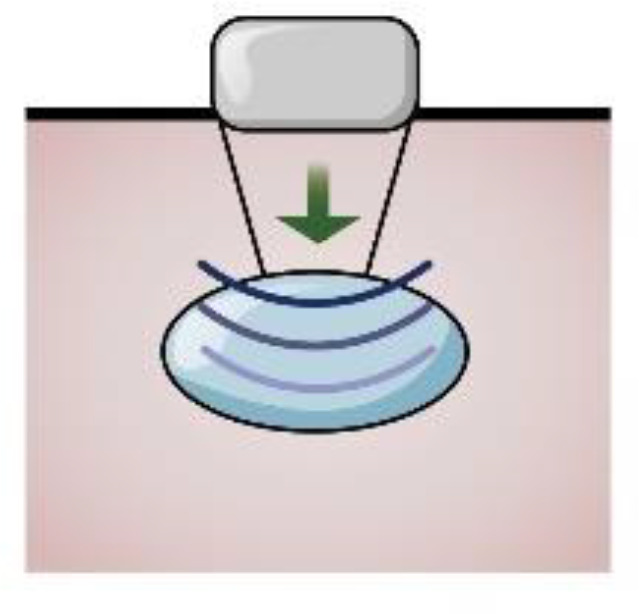
Shear wave speed	shear wave imaging	shear wave speed imaging	Shear wave speed (m/s) Young’s modulus (kPa)	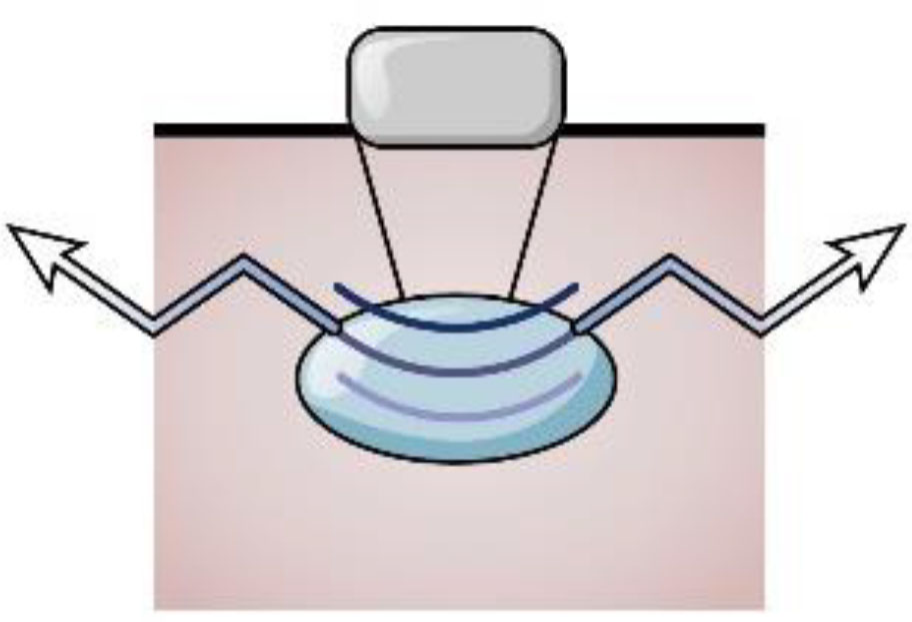
		Transient elastography	Young’s modulus (kPa)	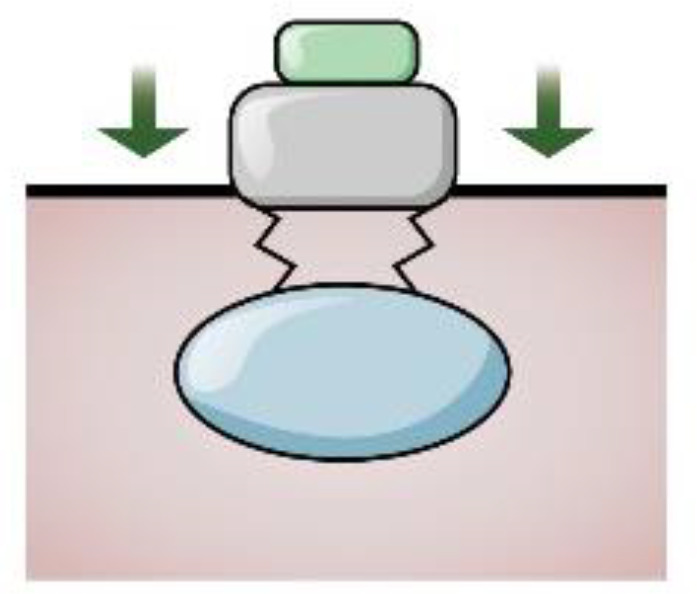

E/B size ratio (ratio of the size of a lesion in the strain image to its size in the B-mode image).

#### 2.2.2 Application of ultrasound elastography in other diseases

Strain imaging has been applied for lesion detection in various tissues (39), such as the auxiliary diagnosis of thyroid nodules (40) and focal pancreatic lesions (41), and has unique advantages in the diagnosis of autoimmune pancreatitis (42). It can also be used to help identify acute and chronic deep vein thrombosis (43) and to assist in the identification of suspicious lymph nodes during lymph node puncture (44). Recent studies have found that strain elastography is also reliable for monitoring relative knee ligament stiffness (45). Sahan MH (46) used strain elastography to assist in measuring cartilage elasticity and evaluating variations in cartilage hardness in the initial phases of OA.

Shear wave imaging is mainly used to diagnose mild fibrosis or cirrhosis (47), and TE has mostly been utilized to assess liver stiffness measures (LSM) in individuals suffering from long-term viral hepatitis or additional illnesses, with more representative results of liver parenchymal stiffness compared to liver biopsy (39). The TE uses an external ‘punching machine’ with controllable vibration to produce shear waves, measure the average shear wave velocity in the region, and convert it into Young’s modulus; hence, the TE standardization technique was specifically used for measuring liver tissue hardness rather than imaging (38). Shear wave elastography based on ARFI techniques can help diagnose the staging of liver fibrosis, detect and characterize focal liver lesions (48), and diagnose benign and malignant thyroid nodules (49), especially in the presence of chronic autoimmune thyroiditis (50). It has also been used for the gastrointestinal tract (51), heart (52), blood vessels, and musculoskeletal (53). Further, it can also be used to improve the accuracy of gastrointestinal tumor staging, assist in making a diagnosis of benign and malignant lymph nodes among individuals with primary cancer, improve the diagnosis of carotid plaque vulnerability (54), evaluate the directional mechanics of the heart and cartilage (52), quantify the mechanical properties of false vocal cords in normal individuals, and evaluate the symmetry of false vocal cords (55). It also has the inherent advantage of diagnosing and treating neurological diseases such as Parkinson’s disease (56), carpal tunnel syndrome (57), chronic stroke (58), and multiple sclerosis (59). Furthermore, it concentrates on the transverse waves created within the tissue, which can be employed for patients with ascites surrounding the liver and is more effective for obese people (60, 61).

#### 2.2.3 Ultrasound elastography distinguishes pathological cartilage from normal cartilage

The health and maintenance of articular cartilage highly depend on appropriate mechanical loading. In animal and human studies, both high loads and low physical activity have led to cartilage thinning and softening (62–64). Due to gravity forces, the body weight load may show different elastography features in normal and pathological conditions compared to the joints of the upper limb. The hardness of cartilage changes before cartilage structure changes in the early stage of KOA (65); hence, it is important to evaluate cartilage elasticity (17). Strain elasticity imaging induces echo signal movement around the tissue, which the probe stresses to exert stable and regular pressure on the target tissue. The strain rate is obtained by contrasting the echo signals prior to and following pressure, where a higher strain (38) ensures more excellent material elasticity.

In a distal femoral cartilage evaluation study, real-time elastography was objectively used to evaluate tissue elasticity, where the diseased cartilage area’s median strain value was substantially greater than healthy cartilage (5). Similarly, another study demonstrated that elastography might be an effective tool for displaying diseased cartilage and being used to distinguish diseased cartilage from normal cartilage (18), where the median strain value of the pathological femoral cartilage area was significantly higher than normal cartilage. In ultrasound elastography, blue coding of normal cartilage tissue shows typical echoless imaging characteristics, which are excellent clarity, devoid of focal defects, smooth bone surface, and unchanged thickness compared with adjacent tissues. In contrast, the pathological cartilage tissue coding showed irregular color changes from blue to red. In an event where US shows no difference in cartilage thickness, real-time elastography can be utilized to determine the change in cartilage hardness by calculating the strain ratio of the region. This technique can be used to forecast the degenerative changes in the knee joint after anterior cruciate ligament reconstruction (66). Shear wave elastography is a reliable, harmless, and acceptable technique for evaluating pathological cartilage (17, 67), where the shear wave value is correlated with the cartilage US score. The faster the shear wave speed or the greater Young’s modulus, the lower the elasticity of the tissue and the higher the hardness. Different hardness levels can identify normal or abnormal tissues (36). Therefore, elastography can be used as an early detection method for evaluating OA cartilage injury (68).

### 2.3 Ultrasound is a reliable tool for quantifying cartilage thickness

For measuring cartilage thickness, US is a dependable, unbiased, and objective technology (6). It includes the measurement of the articular cartilage thickness of the knee, wrist, shoulder, and metacarpophalangeal joint. Measurement of early alterations in femoral cartilage thickness following ACL reconstruction helps evaluate and prevent the occurrence of KOA (29). Articular cartilage thickness of metacarpophalangeal (MCP) and proximal interphalangeal (PIP) joints were measured to assist early identification and monitoring of bone erosion and cartilage injury in rheumatoid arthritis (69), as well as to measure the cartilage thickness of juvenile knee joint to assist the diagnosis of juvenile idiopathic arthritis (70). Prenatal US examination of fetal nasal soft tissue thickness and nasal bone length can effectively reduce the birth rate of fetuses with Down’s syndrome, thus having high accuracy and clinical application value for screening fetuses with Down’s syndrome (71, 72).

Due to mechanical damage, late-stage OA patients are characterized by joint cartilage damage, loss, and thinning of thickness. US recognition of changes in cartilage status is crucial for evaluating the effectiveness of strategies to reduce the risk of development and progression of KOA (4, 10). OA patients with cartilage defects face accelerated progression of OA (19) since cartilage thickness is an essential index for detecting the occurrence and development of OA, where detection and quantification of cartilage thickness and damage are crucial for evaluating OA’s progression and treatment. The main characteristics of articular cartilage are hypoechoic, anechoic, and clear cartilage bone and synovial cartilage interfaces. The high echo lines between the surface of the cartilage and synovial fluid are called “interface signs”. Identifying the cartilage bone and synovial fluid cartilage interfaces is particularly important for measuring cartilage thickness (9, 10). A significant number of studies in the literature suggest that US is a feasible clinical tool for assessing cartilage thickness, which has been found to be consistent with *in vitro* animal studies and autopsy thickness values and highly correlated with cartilage thickness measured by MRI (10, 73). Similarly, it was found *in vivo* that ultrasonography can accurately measure cartilage thickness as well as the scope of damaged cartilage in the knee joint (74). Arthroscopic US can avoid bone occlusion in the joint and thoroughly evaluate the entire cartilage in the joint; in addition, it can accurately measure the thickness of the cartilage in the case of extremely thin cartilage, assessing the degree of regional cartilage damage relative to the thickness of the entire articular cartilage (75). A significant correlation between US and arthroscopy has shown that US has an excellent predictive value in detecting the severity of cartilage degeneration and can also detect early pathological changes in articular cartilage.

Currently, the determination of cartilage thickness using US primarily relies on image segmentation or original radio frequency (RF) signal analysis. Although static US scans provide high-resolution and high-quality images, cartilage data analysis faces challenges due to low contrast, high-level speckle noise, and various imaging issues in US images. There is an urgent need for accurate, stable, and fully automated methods to enhance US images and segment cartilage, thus enhancing the widespread utility of US imaging techniques. Various technologies have been developed to address this need, including multipurpose beta-optimized recursive histogram equalization (MBORBHE), the random walker (RW) algorithm, the local statistical level set method (LSLSM), and deep learning methods (6, 76–78). For instance, MBORBHE is utilized to enhance cartilage regions in US images, preserving essential information such as brightness shifts and contrast enhancement. However, this method may inadvertently enhance the soft tissue interface, potentially affecting cartilage segmentation and thickness measurement. The RW algorithm is employed for automatic cartilage segmentation, although it is susceptible to changes in anatomical structure (6). Another approach involves using the LSLSM to segment cartilage from two-dimensional knee joint US data. While it yields promising results, post-processing of the segmented image using connected component labels is necessary (77). Deep learning frameworks, such as convolutional neural networks, are employed to regress the cartilage interface distance field, delineate cartilage interfaces, and calculate cartilage thickness (76). Furthermore, the original RF signal is tracked using peak detection algorithms to analyze surface displacement and calculate cartilage thickness (78). Active or passive movements during US evaluation can also be used to observe the flow of synovial fluid within focal cartilage defects that are almost invisible in static US imaging, significantly improving the sensitivity and specificity of the examination. ACL injury is a key risk factor for the development of KOA, and imaging of this ligament under static US is difficult, but dynamic US can help confirm structural lesions. Dynamic US can help better visualize and simulate different anatomical structures in daily life, which is helpful for the diagnosis of OA complications such as synovitis and joint effusion. It plays an increasingly important role in evaluating joint cartilage tissue (79, 80) (**Table 4**).

**Table 4:** Study on the measurement of cartilage thickness by ultrasound.

Authors	Material	Transducer frequency (MHz)	Anatomical site	n	Studies	Conclusion
Yagi M, Taniguchi M, et al. (11)	OA patients	5- 18	MFC	22	22	Ultrasound can objectively and quantitatively evaluate early cartilage degeneration in osteoarthritis.
Okada S, Taniguchi M, et al. (81)			MFC	126	118	
Okada S, Taniguchi M, et al. (82)		44	MFC	56	34	
Lisee C, Harkey M, et al. (29)	Patients 4 to 6 months after ACLR	12	FAC	20	120	Ultrasound can diagnose thickening and thinning of femoral cartilage.
Pradsgaard DO, Fiirgaard B, et al. (70)	Children with JIA	6-14	FAC	23	138	Ultrasound can be consistent with MRI in measuring the average thickness of cartilage.
Printemps C, Cousin I, et al. (83)	4–12-week-old infants	8-10	Pubic cartilage	948	1896	Ultrasound can improve treatment decisions for hip dysplasia by measuring cartilage thickness.
Desai, P. Hacihaliloglu, I. et al. (6)	Healthy volunteers	8- 12	FAC	10	80	Ultrasound can objectively and effectively evaluate cartilage thickness.
Harkey MS, Blackburn JT, et al. (84)		12	MFC	25	75	
Devrimsel G, Beyazal MS, et al. (75)		7- 12	FAC	30	180	
Güvener O, Dağ F, et al. (12)		5-13	FAC	16	192	
Yildirim A, Onder ME, et al. (85)		7 - 12	FAC and talar cartilage	55	440	
Moller B, Bonel H, et al. (86)	RA patients	10 –15	MCP and PIP finger joint cartilage	48	1152	Ultrasound can distinguish early RA from healthy joints by reducing cartilage.
Yildirim A, Onder ME, et al. (85)		7 - 12	FAC and talar cartilage	55	440	
Kaya A, Kara M, et al. (87)	SLE patients	7- 12	FAC	29	174	Ultrasound can effectively and reliably evaluate the thickness of femoral cartilage in patients with systemic lupus erythematosus.
Güvener O, Dağ F, et al. (12)	Flatfoot patients	5-13	FAC	16	192	Ultrasound can measure the thickness of distal femoral cartilage in different conditions.
Naredo E, Acebes C, et al. (10)	Body knee specimens	14	FAC	8	24	Ultrasound can more accurately measure normal to moderately damaged cartilage.
Devrimsel G, Beyazal MS, et al. (75)	Patients with hypothyroidism	7- 12	FAC	40	240	Ultrasound can assist in the early diagnosis of osteoarthritis in patients with hypothyroidism.

MFC, medial femoral condyle; FAC, femoral articular cartilage; ACLR, anterior cruciate ligament reconstruction; JIA, juvenile idiopathic arthritis; MCP, metacarpophalangeal; PIP, proximal interphalangeal; RA, rheumatoid arthritis; SLE, systemic lupus erythematosus; US, ultrasound; MRI, magnetic resonance imaging.

## 3 Conclusion and future perspectives

US has many characteristics that make it valuable in evaluating OA cartilage damage. Quantitative ultrasound parameters can detect early collagen fracture of articular cartilage and rough and uneven articular cartilage surfaces, which can be utilized to assess the integrity of articular cartilage and provide helpful information for quantitative ultrasound diagnosis of early OA. As a novel ultrasonic imaging method, elastography is more promising than traditional US, where additional imaging can provide valuable information on articular cartilage elasticity to clinicians. Elastography also finds applications in determining tissue properties, structure, and function. Currently, it is shown in the initial rhinoplasty and revision nasal surgery that strain ultrasound elastography can assist in the selection of the correct tissue for cartilage transplantation. It is foreseeable that more advanced US technologies will continue to rapidly evolve in the coming years. US can accurately measure cartilage thickness, degree, and depth of cartilage defects, enhance accuracy in clinical OA classification, and improve and evaluate OA’s progress and treatment response by detecting and monitoring the therapeutic effect of cartilage injury. In recent times, there has been a pervasive utilization of three-dimensional US imaging technology, addressing the constraints associated with two-dimensional imaging for the observation of cartilage’s three-dimensional structure. This advancement facilitates comprehensive and volumetric cartilage imaging, furnishing practitioners with augmented data for precise morphological and functional assessments. Moreover, the ongoing evolution of artificial intelligence (AI) technology within the realm of US cartilage imaging, encompassing the training of deep learning models, holds promise for automating the analysis and diagnosis of cartilage imaging data, thereby enhancing diagnostic precision and workflow efficiency. US is a rapidly growing technology with enormous possibilities for future clinical applications. We have shown in our overview that US can be employed in basic studies of articular cartilage to evaluate early histopathology, elasticity, thickness, degree of changes, and defects in articular cartilage, which play a crucial role in detecting early bone and joint disorders. Although there are still many challenges in the development of US diagnostic tools, they play an increasingly important role in the diagnosis of cartilage injuries.
